# Whole-Body Vibrations Associated With Alpine Skiing: A Risk Factor for Low Back Pain?

**DOI:** 10.3389/fphys.2018.00204

**Published:** 2018-03-09

**Authors:** Matej Supej, Jan Ogrin, Hans-Christer Holmberg

**Affiliations:** ^1^Faculty of Sport, University of Ljubljana, Ljubljana, Slovenia; ^2^School of Sport Sciences, UiT Arctic University of Norway, Tromsø, Norway; ^3^School of Kinesiology, University of British Columbia, Vancouver, BC, Canada; ^4^Department of Physiology and Pharmacology, Karolinska Institutet, Stockholm, Sweden

**Keywords:** biomechanics, injury prevention, kinematics, kinetics, recreational skiing, shock, ski racing

## Abstract

Alpine skiing, both recreational and competitive, is associated with high rates of injury. Numerous studies have shown that occupational exposure to whole-body vibrations is strongly related to lower back pain and some suggest that, in particular, vibrations of lower frequencies could lead to overuse injuries of the back in connection with alpine ski racing. However, it is not yet known which forms of skiing involve stronger vibrations and whether these exceed safety thresholds set by existing standards and directives. Therefore, this study was designed to examine whole-body vibrations connected with different types of skiing and the associated potential risk of developing low back pain. Eight highly skilled ski instructors, all former competitive ski racers and equipped with five accelerometers and a Global Satellite Navigation System to measure vibrations and speed, respectively, performed six different forms of skiing: straight running, plowing, snow-plow swinging, basic swinging, short swinging, and carved turns. To estimate exposure to periodic, random and transient vibrations the power spectrum density (PSD) and standard ISO 2631-1:1997 parameters [i.e., the weighted root-mean-square acceleration (RMS), crest factor, maximum transient vibration value and the fourth-power vibration dose value (VDV)] were calculated. Ground reaction forces were estimated from data provided by accelerometers attached to the pelvis. The major novel findings were that all of the forms of skiing tested produced whole-body vibrations, with highest PSD values of 1.5–8 Hz. Intensified PSD between 8.5 and 35 Hz was observed only when skidding was involved. The RMS values for 10 min of short swinging or carved turns, as well as all 10-min equivalent VDV values exceeded the limits set by European Directive 2002/44/EC for health and safety. Thus, whole-body vibrations, particularly in connection with high ground reaction forces, contribute to a high risk for low back pain among active alpine skiers.

## Introduction

Although physical activity is beneficial to human health, for example by reducing the risk of chronic disease, among the most common injuries in modern Western societies are those related to sports (Parkkari et al., 2001). For instance, alpine skiing is associated with high rates of injury for both recreational and competitive athletes (Hunter, 1999; McBeth et al., 2009; Hebert-Losier and Holmberg, 2013; Soligard et al., 2015; Stenroos and Handolin, 2015; Weber et al., 2015; Haaland et al., 2016; Müller et al., 2016; Supej et al., 2016). In addition, problems caused by overuse are also recurrent in alpine skiing, with low back pain (LBP) being the most common (Hildebrandt and Raschner, 2013; Spörri et al., 2015; Supej et al., 2016).

It has been proposed that such overuse injuries to the lower back might be reduced by controlling and/or reducing frontal and lateral bending, as well as torsion of the trunk and peak load while skiing (Spörri et al., 2015, 2016). Moreover, these studies found no differences in low-back kinematics when skis with different side-cut radii were utilized. Most of the underlying deteriorations of the spine develop early in the career of the alpine skier (Rachbauer et al., 2001), when on-snow training is not usually performed on courses resembling those used in competitions. Nevertheless, little is presently known about the nature and frequency of overuse injuries in alpine skiing, including when and why they occur (Supej et al., 2016).

On the other hand, exposure to whole-body vibrations (WBV) in connection with various occupations is strongly related to low back pain (Hulshof and van Zanten, 1987; Bovenzi and Hulshof, 1999; Lings and Leboeuf-Yde, 2000; Burström et al., 2015), which is one reason for the establishment of international health and safety standards by ISO 2631:1997 (ISO, 1997) and the European Directive 2002/44/EC (EU, European Parliament and the Council of the European Union, 2002) in this context (Griffin, 2004).

Commonly, the dynamic response of the body of an individual seated or standing still to vibrations is expressed in terms of mechanical impedance or apparent mass (i.e., the ratio between motion and force at the driving point) and transmissibilities (i.e., the ratio between two motions at distant points) (Matsumoto and Griffin, 1998). Recent studies have demonstrated that when standing still while barefoot or wearing regular shoes without any additional load, the dynamic response to vibrations (apparent mass) depends on posture (Subashi et al., 2006, 2008; Tarabini et al., 2013). More specifically, acceleration of higher frequencies at the driving point were found to be significantly more attenuated within the body with the knees bent than when erect. Nevertheless, higher spinal loads were caused by low and higher frequency WBV in both of these postures (Rohlmann et al., 2014).

During slalom and giant slalom ski racing, the most powerful vibrations had a frequency of less than 30 Hz, with the root mean square vibrational values being higher in the case of giant slalom (Spörri et al., 2017). In addition, that investigation suggested that vibrations of lower frequencies, i.e., between 4 and 10 Hz, might be particularly prone to cause injuries of the back in connection with alpine ski racing.

Furthermore, one beginner and one skilled skier skiing under uncontrolled conditions were found to be exposed to WBV that exceeded the 2-h equivalent values set by the European Directive 2002/44/EC (Tarabini et al., 2015), but no generalization could be made due to the small sample size. Therefore, it remains to be determined which forms of skiing (e.g., skidding, carved turns) are more likely to produce vibrations and whether these exceed the thresholds set by the ISO standard and European Directive. The current investigation was designed to answer these questions.

## Methods

### Measurements and collection of data

Eight highly skilled ski instructors, all former competitive racers, performed six different types of skiing on 165-cm slalom/carving skis (SLX, Elan d.d., Begunje, Slovenia) with a 14.5-m side-cut radius. These skis complied with International Ski Association (FIS) regulations for slalom. These six types of skiing corresponded to the core stages of progression typically followed by ski instructors:

1. *Straight running*: From a stationary start, skiing straight downhill for ~40 m in a basic stance.
2. *Plowing*: After achieving straight running speed, slowing down by plowing until coming to a complete stop.
3. *Snow-plow swinging*: Making consecutive turns in the snow-plow position.
4. *Basic swinging*: Making turns slowly with the skis parallel.
5. *Short swinging*: Making “slalom-like” turns rapidly with the skis parallel.
6. *Carved turns*: Making long, wide (carving) turns with the skis parallel and without skidding to the side.

It should be noted that snow-plowing, as well as basic and short swinging by definition involve skidding, where the ends of the skis glide out to the side; while with carving turns the tip and end of the ski follow the same trajectory. The ski course for testing was well prepared and groomed, the snow natural and well packed, and the air temperature between −3 and −7°C with partially sunny weather that provided good visibility.

An integrated electronic piezoelectric accelerometer (sensitivity: 100 mV/g, range: 50 g, mass: 4.3 g) (3097A2, Dytran Instruments Inc., Chatsworth, CA, USA) was firmly attached to each ski boot to record accelerations of the superior-inferior axis. In addition, three variable capacitance accelerometers (sensitivity: 80 mV/g, range: 50 g, mass: 12 g) (7300A5, Dytran Instruments Inc., Chatsworth, CA, USA) attached to a belt tightened around and taped to the body measured accelerations of the sacrum in three dimensions aligned with the orientation of the trunk. A 10-Hz Global Navigation Satellite System (GNSS) (ST 1612 G, Locosys Technology Inc., Taipei, Taiwan) with external antennae tracking both United States (GPS) and Russian (GLONASS) satellites (1240, Locosys Technology Inc., Taipei, Taiwan) was positioned at the level of the upper thoracic spine (T2–T4) to track the skier's speed. These accelerometers and the GNSS were wired to a 24-bit, 200-kHz data acquisition system (DEWE-43 & DEWESoft X2, DEWESoft d.o.o., Trbovlje, Slovenia).

The data on accelerations, sampled at 5 kHz, were used to calculate power spectrum densities (PSD) and ground reaction forces, while the positioning data allowed monitoring of speed. The accuracy and tolerance of the entire set-up for determining WBV adhered to the requirements of the ISO 8041:2005 (ISO, 2005). The study design was pre-approved by the Regional Ethics Committee of the University of Ljubljana and informed written consent obtained from all the volunteers prior to testing.

### Computation of the parameters

To determine the frequencies of WBV, the single-sided non-parametric Fast-Fourier-Transform was first used to calculate power spectrum densities (PSD) from the ski boot accelerations for each skier and each type of skiing. The average PSD curves for both legs of each skier were created and then the data for all participants while performing the same type of skiing were combined to calculate the average power spectrum. For more effective illustration, the PSD graphs presented display a logarithmic scale and have been smoothed with an equally-weighted, zero-lag moving average filter with a length of five.

Further evaluation of WBV was based on the approach described in ISO 2631-1:1997 and in accordance with the European Directive 2002/44/EC. The raw data transmitted from the principal surface supporting the accelerometers on the ski boots were first bandpass-filtered from 0.5 to 80 Hz. Subsequently, frequency weighting of these accelerations in combination with the multiplier for a vertical z-axis for a standing position were applied, as required by the ISO 2631-1:1997 standard, to calculate the following exposures to periodic, random or transient vibrations:

1. the weighted root-mean-square acceleration (RMS):
  $\text{RMS}=\;{\left[\frac{1}{\text{T}}\int_{0}^{\text{T}}{\text{a}}_{\text{w}}^{2}\left(\text{t}\right)\text{dt}\right]}^{\frac{1}{2}}$, where T was the duration of measurement and a_w_ acceleration weighted as a function of time t
2. the crest factor (CF), defined as the modulus of the ratio of the maximum instantaneous peak value of the frequency**-**weighted acceleration to its RMS value,
3. the maximum transient vibration value (MTVV), i.e., the running RMS, given as the maximum in time of RMS(t_0_):
  $\text{RMS}\left({\text{t}}_{0}\right)=\;{\left[\frac{1}{\tau }\int_{{\text{t}}_{0}-\tau }^{{\text{t}}_{0}}{\text{a}}_{\text{w}}^{2}\left(\text{t}\right)\text{dt}\right]}^{\frac{1}{2}}$, where τ was the integration time (according to the ISO recommendation that τ = 1 s) and t_0_ the time-point of observation
4. the fourth-power vibration dose value (VDV):
  $\text{VDV}=\;{\left[\int_{0}^{\text{T}}{\text{a}}_{\text{w}}^{4}\left(\text{t}\right)\text{dt}\right]}^{\frac{1}{4}}$.

From each type of skiing involving turning and each skier, the first and last turn were excluded from the analysis, resulting in ~15-s periods of data collection. In accordance with the standards, vibrations from both ski boots were considered. As required by the standard, the VDV values were expressed as 8-h and 10-min exposures for direct comparison to the action and limit values formulated in the European Directive 2002/44/EC (Griffin, 2004), while the RMS values were compared to the action and limit exposure values set by this same directive.

The three variable capacitance accelerometers positioned at the pelvis allowed estimation of ground reaction forces (GRF) as multiples of body weight (BW). These calculations involved the assumptions that the pelvis was the center of mass and air drag negligible. The GRF values were smoothed with a zero-lag third-order digital Butterworth filter employing a cut-off frequency of 7 Hz. Thereafter, the peak GRF values for turns were calculated and utilized for further evaluation. From the GNSS data mean skiing speeds were calculated. All calculations were performed with the DEWESoft X2 and Matlab 7.7 software (Mathworks Inc., Natick, MA, USA).

### Statistical analyses

The means and standard deviations for all parameters are presented. The Shapiro-Wilk test was used to explore the normality of distributions and, when necessary, the Box-Cox power transformation was performed to achieve normality. One-way ANOVA with repeated measures was used to test for differences between parameters. Mouchly's *W*-test was used to indicate whether the assumption of sphericity had been violated and, if so, this was corrected for with the epsilon value, utilizing either the Huynh-Feldt or Greenhouse-Geisser procedure. For *post-hoc* analysis, paired sample *t*-tests were applied to test for differences between parameters. The false discovery rate for a family of hypotheses was controlled for by the Benjamini–Hochberg–Yekutieli procedure (Benjamini and Yekutieli, 2001). The level of statistical significance was set at *p* < 0.05 and all statistical analyses carried out with the Matlab software.

## Results

### Whole-body vibrations

**Figure 1:**
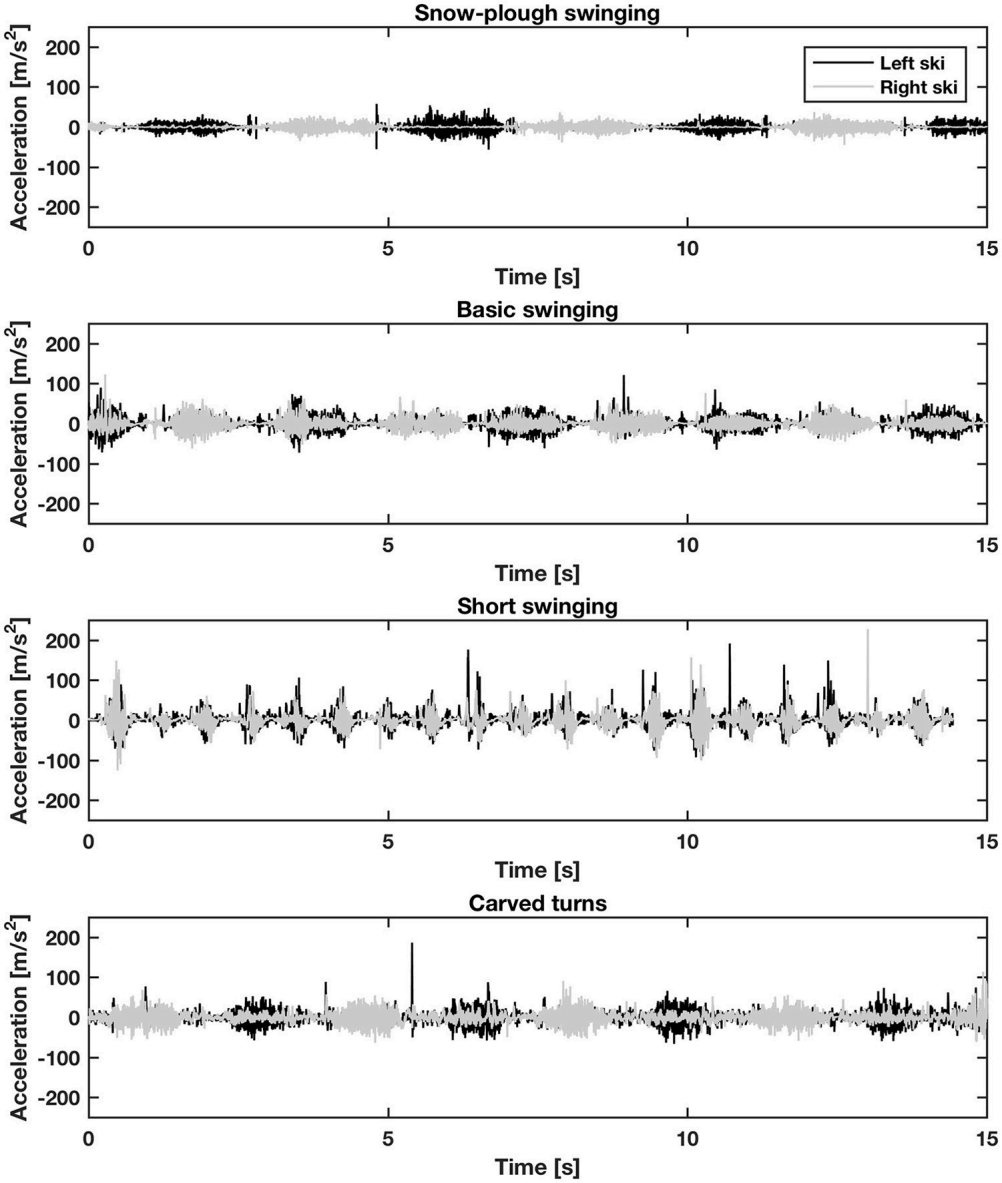
Representative time-courses of acceleration measured at the left and right ski boot of one skier while performing forms of skiing that involved turning.

**Figure 2:**
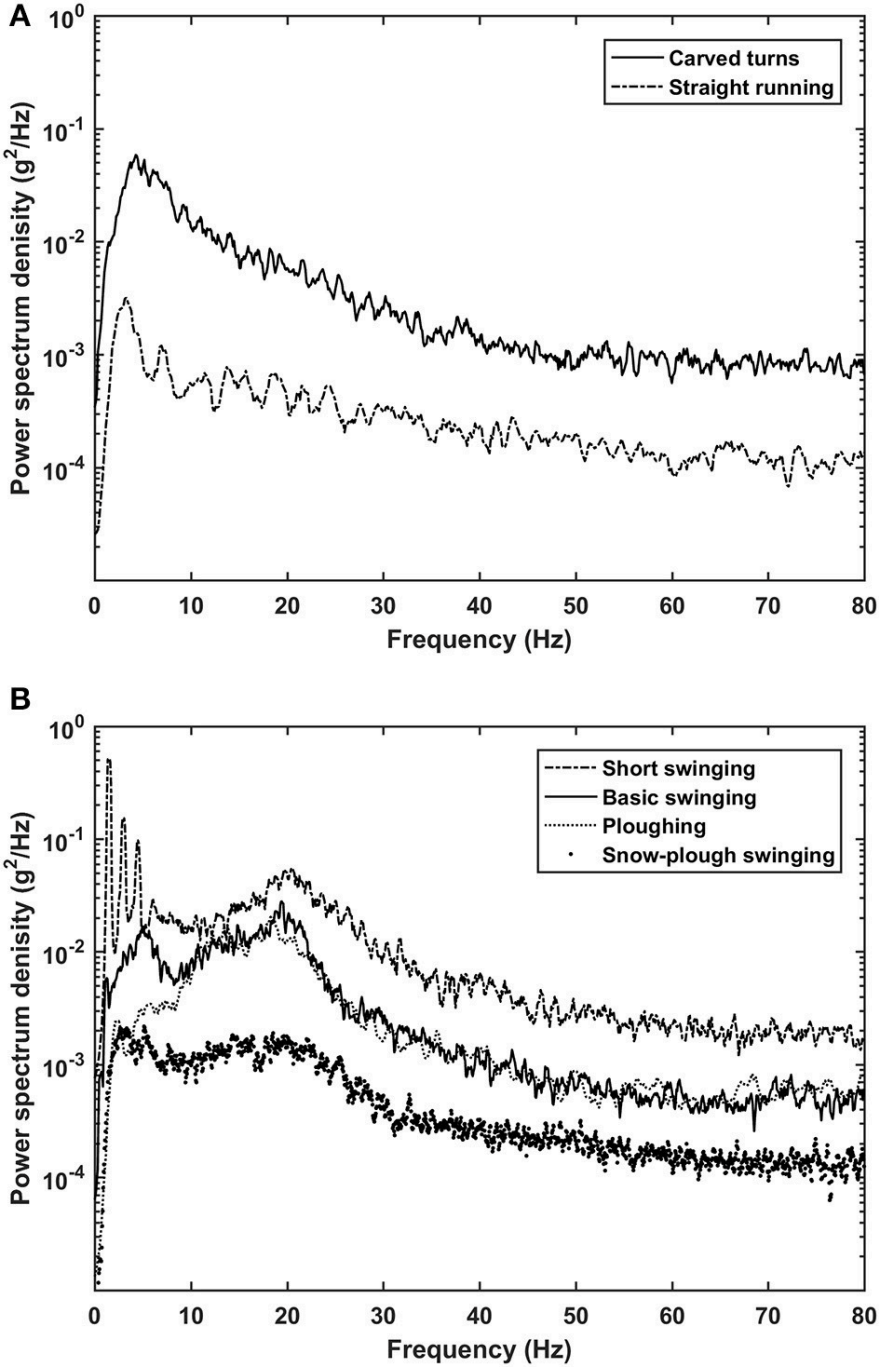
The power spectrum densities for whole-body vibrations associated with carved turns and straight running **(A)**, as well as short swinging, basic swinging, plowing and snow-plow swinging **(B)**.

Representative raw time-courses for acceleration at the ski boots of one subject while skiing with sub-techniques that involved turning are presented in Figure 1 and the overall average PSD values for frequencies up to 80 Hz for the six types of skiing examined in Figure 2. Straight running and carved turns demonstrated similar patterns (Figure 2A), with highest densities between ~3 and ~8 Hz and continuous attenuation at increasing frequencies. At the same time, plowing, snow-plow swinging, basic swinging and short swinging exhibited two regions of intensified PSD (Figure 2B), the first between ~1.5 and ~8 Hz and the second between ~8.5 and ~35 Hz, above which the PSD values declined. These attenuations of the PSD curves continued for all six types of skiing until ~70 Hz, above which the values remained steady until 200 Hz, followed by another attenuation (~200–500 Hz), finally remaining more or less constant until 2.5 kHz, where the power densities were 2–3 orders of magnitude lower than the maximal values.

Evaluation of the exposure to periodic, random and transient WBV associated with all six forms of skiing are also shown in Table 1. In the case of MTVV, one subject had considerably higher values (outliers) for snow-plow swinging and short swinging and had to be eliminated from evaluation of these particular forms of skiing, in order to achieve normality and sphericity of the data. Overall, the highest levels of exposure were observed in connection with short swinging followed by carved turns and the lowest with snow-plow swinging and straight running. The RMS and VDV values for plow were also high, but not the MTVV.

**Table 1:** Comparison of the mean weighted root-mean-square accelerations (RMS, *n* = 8), the fourth-power vibration of doses (VDV, *n* = 8) and maximum transient vibration value (MTVV, *n* = 7) for the six types of skiing.

**Parameter**	**Straightrunning**	**Plowing**	**Snow-plowswinging**	**Basicswinging**	**Shortswinging**	**Carvedturns**	**ANOVA Fstatistics**	***p*-valuesphericity**	**Pairedsample *t*-tests^§^**
RMS	2.55 ± 0.70	6.05 ± 1.38	2.78 ± 0.25	7.73 ± 0.50	12.92 ± 1.49	9.69 ± 0.63	147.06	0.0002	SR/SPS: 0.40
VDV 10 min	22.19 ± 6.69	44.80 ± 11.97	21.44 ± 3.49	56.77 ± 4.98	96.00 ± 13.01	67.48 ± 5.42	92.48	0.0054	SR/SPS: 0.78
VDV 8 h	58.41 ± 17.61	116.10 ± 31.50	56.44 ± 9.17	149.43 ± 13.10	252.67 ± 34.24	177.62 ± 14.27	92.48	0.0054	SR/SPS: 0.78
MTVV^#^	5.11 ± 1.71	7.85 ± 2.63	5.29 ± 0.49	13.11 ± 1.38	19.18 ± 2.06	14.99 ± 1.20	78.17	0.6284	SR/SPS: 0.79

*Overall means ± standard deviations and the results of the ANOVA and paired sample t-test are presented*.

10 min, 10-min equivalent; 8 h, 8-h equivalent;

§ , only pairs for which p ≥ 0.05 are shown; SR/SPS, straight running vs. snow-plow swinging;

# *, one skier was excluded from the MTVV analysis to obtain normal distribution of these data*.

The ISO 2631:1997 states that if CF>9, then not only RMS, but also VDV and MTVV should be taken into consideration. The mean crest factors for straight running and snow plowing exceeded the ISO safety margin, while the maximal CF values for all six types of skiing except plowing also exceeded this margin (Table 2).

**Table 2:** Crest factors for all six forms of skiing.

**Crest factor**	**Straight running**	**Plowing**	**Snow-plow swinging**	**Basic swinging**	**Short swinging**	**Carved turns**
Mean	9.60	6.80	9.58	7.52	8.52	7.60
*SD*	1.67	1.12	1.97	1.13	1.37	1.23
Minimum	6.05	5.36	7.75	6.29	7.02	5.46
Maximum	11.04	8.49	13.70	9.86	10.86	9.05

*SD, standard deviation*.

### Speed and ground reaction forces

**Figure 3:**
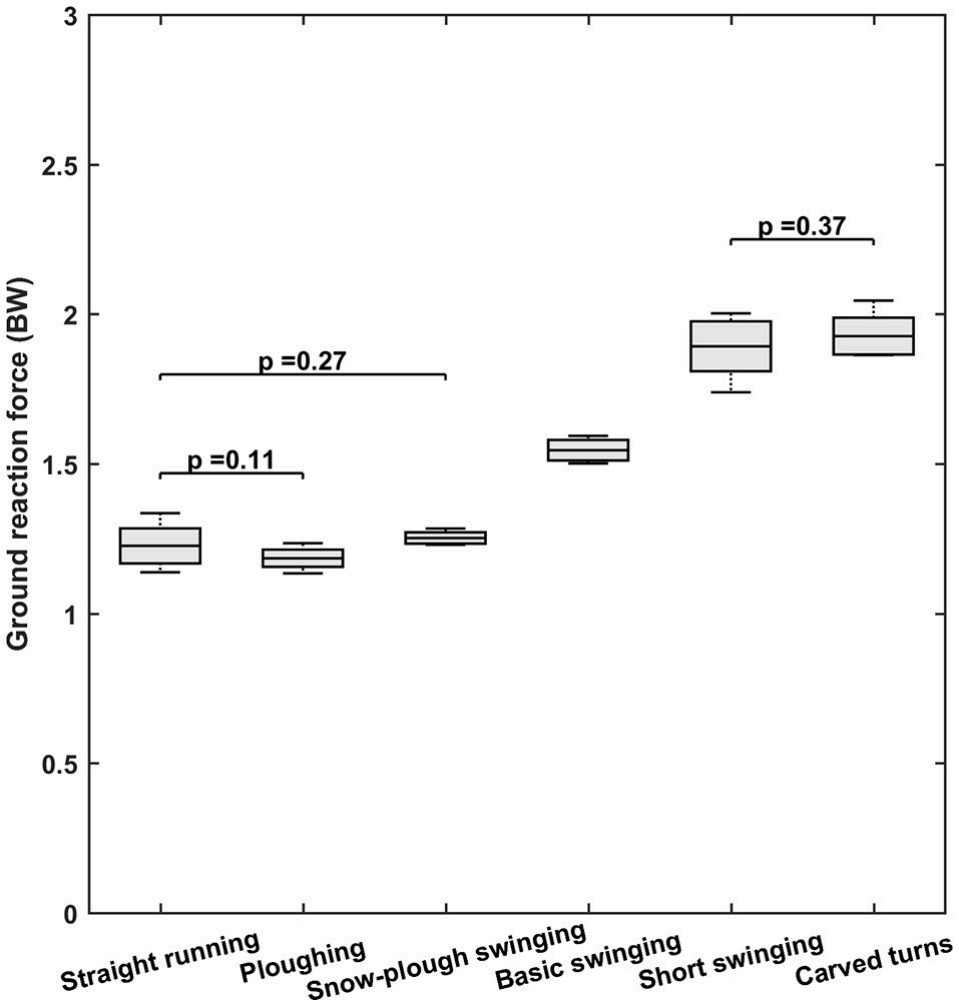
Peak ground reaction forces (GRF) for the six different types of skiing. In each box, the central line indicates the mean and the bottom and top edges the standard deviation. The whiskers extend to the maximal and minimal data points. Note that for more effective presentation, the *p*-values for pairs that did not differ significantly are the only ones shown.

**Figure 4:**
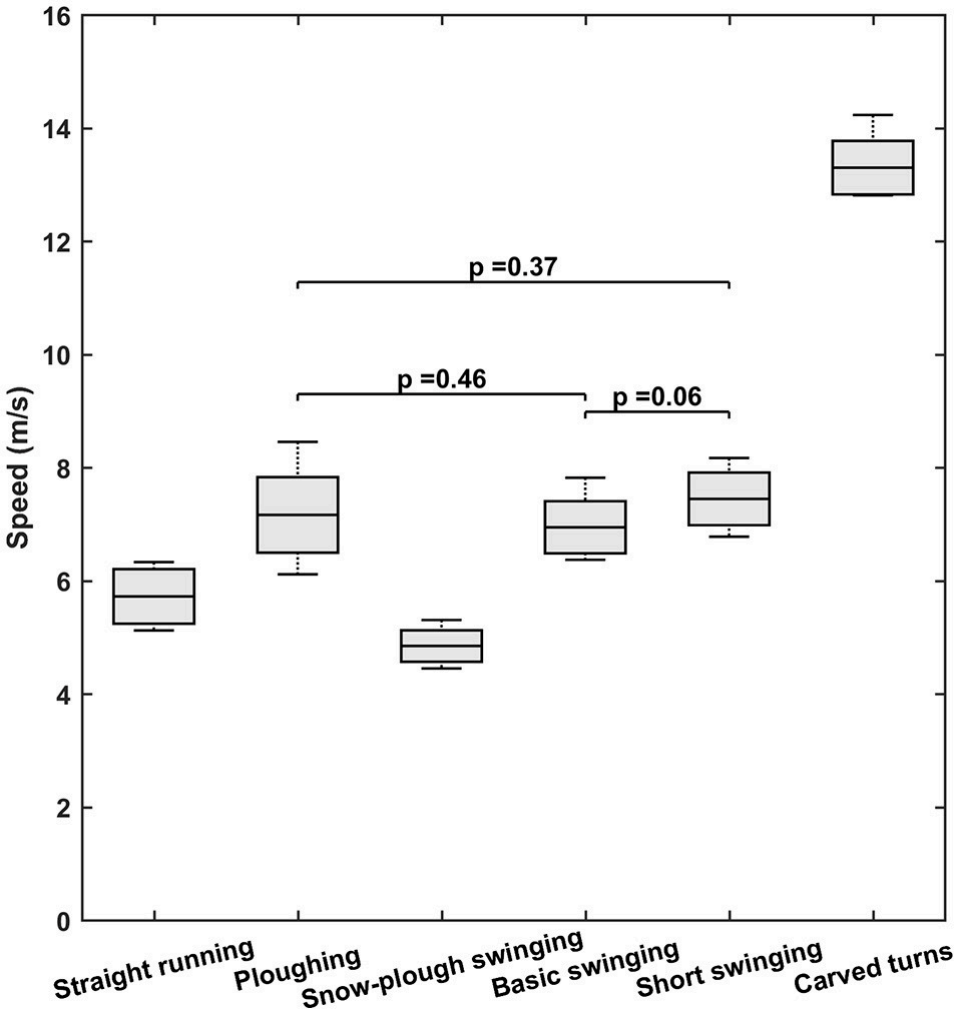
Speeds with the six different types of skiing. In each box, the central line indicates the mean and the bottom and top edges the standard deviation. The whiskers extend to the maximal and minimal data points. Note that for more effective presentation, the *p*-values for pairs that did not differ significantly are the only ones shown.

The mean values and standard deviations for speed and peak GRF are documented in Figures 3, 4, respectively. In these cases, the Shapiro-Wilkinson test confirmed that all data were distributed normally and the condition of sphericity was also satisfied (in the case of speed data after appropriate correction). Application of paired *t*-tests revealed that the peak GRFs for straight downhill and plowing did not differ significantly (*p* = 0.11), as was also the case for plowing vs. snow-plow swinging (*p* = 0.27) and basic swinging vs. carved turns (*p* = 0.37). Comparison of all other possible pairs demonstrated significant differences (*p* < 0.0001). Overall, the mean GRFs were lowest for straight running (1.23 BW) and plowing (1.18 BW) and highest for short swinging (1.89 BW) and carved turns (1.93 BW).

With respect to speed, plowing and carved turns were associated with means that differed significantly from all the other types of skiing, while the mean values for straight downhill, basic and short swinging did not differ from each other. The lowest mean speed was observed in connection with snow-plow swinging (4.8 m/s) and the highest with carved turns (13.3 m/s).

## Discussion

The major novel findings of the present investigation were as follows: (i) all types of skiing examined produced whole-body vibrations (WBV), with the highest power spectrum densities (PSD) ranging from ~1.5–8 Hz; (ii) intensified PSD between 8.5 and 35 Hz was observed only with the types of skiing that involved skidding; (iii) the RMS values for 10 min of short swinging and carved turns and all 10-min equivalent VDV values exceeded the limit values formulated by the European Directive 2002/44/EC for health and safety; and, finally, (iv) measurement of the WBV, particularly in connection with high ground reaction forces, revealed an important high-risk factor for low back pain in active alpine skiers.

### Whole-body vibrations associated with different forms of alpine skiing

Our present findings demonstrate that all forms of alpine skiing produce vibrations. The WBV in the PSD spectrum below 8 Hz was associated with absence of both turning and skidding (straight running), presence of skidding (snow-plow swinging and short swinging), as well as carved turns. At the same time, higher frequency vibrations (8–35 Hz) were intensified only with skiing techniques that by definition involved side**-**skidding, in line with a previous pilot study (Supej, 2013). Furthermore, the regions of intensified and attenuated power spectrum densities associated with skidding here were similar to those reported for slalom and giant slalom ski racing (Spörri et al., 2017). This indicates that even competition skiing involves skidding, despite the fact that elite athletes strive for carving turns.

Interestingly, neither the peak PSD values for the low-frequency vibrations associated with all skiing forms nor with the “skidding vibration” were centered around the first two typical eigen-frequency values reported previously for skis, i.e., *f*_1_ = ~10–13 Hz and *f*_2_ = ~40–50 Hz (Piziali and Mote, 1972; Fischer et al., 2007). This discrepancy has two interesting implications: first, the frequencies measured here are not caused by the ski's own chattering, but rather by movements (e.g., turning, skidding) during skiing. Secondly, in order to optimize performance and kinaesthetic feeling, manufacturers appear to have developed skis with properties that avoid resonances in the two most dominant frequency ranges of the PSD.

### Comparison of the whole-body vibrations associated with alpine skiing to recommendations for health and safety

In comparison to the 8-h limit values set by the European Directive 2002/44/EC for health and safety, the RMS and 8-h equivalent VDV values here were 2–11- and 49–220-fold higher, respectively (Table 1). However, a typical descent from a ski lift or racing run lasts ~1 min and most skiers perform 10 or more runs daily, making such comparisons to 8-h exposures somewhat problematic. On the other hand, even comparison to 10-min equivalents (Griffin, 2004) revealed that short swinging and carved turns exceeded the limit values, while basic swinging was close to this limit and plowing exceeded the action value (Table 1). For example, 10 min of short swinging was found to result in exposure to vibration equivalent to ~18 min of carved turns or 216 min of snow-plow swinging. The 10-min equivalent VDV values here were ~7–32-fold higher than the corresponding limits.

These observations reveal that WBV constitute an important risk factor for LBP in alpine skiers (Seidel and Griffin, 2001; Burström et al., 2015), particularly since substantial acceleration of the spine typically occurs between 4 and 10 Hz, during alpine skiing as well (Kiiski et al., 2008; Spörri et al., 2017). Interestingly, the average 10-min equivalent VDV values for plowing and basic swinging obtained here corresponded closely to the 2-h equivalent VDV values (scaled to 10-min equivalent values for comparison) reported previously for those two skiers under uncontrolled conditions (Tarabini et al., 2015).

Although the vibrations with all skiing forms examined here exceeded the WBV threshold limits for safety, there were substantial differences between these forms in this respect. Importantly, carved turns involved significantly less WBV than short swinging and only slightly more than basic swinging, the first and simplest form of “parallel skiing.” Even though our measurements were performed during “free skiing” by former competitive ski racers, this observation should probably be taken into account when regulations concerning equipment, slope preparation and course setup are formulated with the aim of making competitive skiing safer.

Due to random bumps on uneven terrain, transient vibrations (occasional shocks or short-term vibrations) are also to be expected during alpine skiing. Indeed, these occurred in all forms of skiing investigated here, particularly during short swinging (Figure 1). However, the mean crest factor values here (Table 2) were not as high as expected. Surprisingly, only in the cases of straight running and snow-plow swinging did these values exceed the margin (CF > 9 according to ISO 2631-1:1997) above which evaluation of VDV and MTVV to verify exposure to WBV is obligatory. This reflected the fact that for all other types of skiing, the RMS values were so high that the ratio of the maximal instantaneous peak value of the frequency weighted acceleration signal to the corresponding RMS value (i.e., CF) remained below the threshold. Note that the latter situation, in combination with the high VDV and MTVV values observed, demonstrate clearly that our skiers were actually exposed to both short**-** and longer-term vibrations.

### Ground reaction force and speed

Peak resultant forces on the spine are on the average 24% higher in the presence than absence of vibrations (Rohlmann et al., 2014). Therefore, the high GRF values observed both here and earlier in connection with competitive skiing (Supej et al., 2004, 2015; Supej and Holmberg, 2010; Vaverka and Vodickova, 2010; Spörri et al., 2015, 2016), in combination with intensive WBV (Burström et al., 2015), support the conclusion that vibrations are at least partially responsible for the high incidence of low back pain in alpine skiers. In addition, alpine skiing typically involves relatively extensive flexion of the hip joint, during which muscle forces, while maintaining trunk equilibrium, increase compression and shear forces on the spine substantially (Seidel and Griffin, 2001; Wang et al., 2010).

On the other hand, flexion, particularly at the knee joint, exerts an important influence on apparent mass behavior in response to WBV (Subashi et al., 2006, 2008; Tarabini et al., 2013). More specifically, the resonance frequency is reduced significantly as the knees become more bent. The static conditions employed in previous studies, with no additional load and standing either barefoot or in everyday shoes, differ considerably from those encountered during alpine skiing, thus, future studies should be designed to elucidate the effect of apparent mass in this context.

From our current findings, speed itself does not appear to have contributed directly to the WBV, since the highest exposure was observed even at quite low speeds during short swinging and, on the other hand, the highest speeds during carved turns were associated with substantially lower WBVs than short swinging. This is somewhat contradictory to the previous findings on one beginner and one skilled skier (Tarabini et al., 2015). More systematic investigations in the future will help to further elucidate the connection between WBV and speed.

### Methodological considerations

It was challenging to position the accelerometers here for measurement of WBV during alpine skiing. Nevertheless, the ISO 2631-1:1997 specifies that the transducers should be positioned so as to indicate the vibration at the interface between the human and source of vibration, or more specifically, measurements on the feet should be made at the surface where the feet are most supported. From this perspective, positioning on the ski boots, although not immediately obvious, was fully in line with this standard.

It is not yet known whether the ISO 2631-1:1997 and European Directive 2002/44/EC recommendations for health and safety are also appropriate for sports. However, these threshold values were set for occupations involving standing-up, while not necessarily stationary or fully extended, as well as when bearing heavy loads. This is undeniably similar to the situation during alpine skiing and exposure of active alpine skiers, including competitors, to WBV (e.g., days per year) is comparable to that associated with certain occupations covered by the standard and the directive.

Estimation of the ground reaction forces using accelerometers here involve the assumption that the pelvis was the center of mass and may therefore be somewhat biased. The basic concepts of Newtonian mechanics dictate that the reliability of our estimation of the GRF on the basis of a single-point acceleration depends on the extent to which this acceleration matches the “model acceleration” of the center of mass. Since the largest contribution to the GRF during the alpine skiing turns can be attributed to the radial forces and body flexion-extension, for the purpose of this study this estimation of overall load was considered to be sufficient.

Finally, since our measurements were performed on a moderate incline under nearly ideal conditions of snow and weather, generalization is not straightforward. However, it can be speculated that, for example, icy conditions and/or more difficult slopes would result in more vigorous WBV.

## Future perspectives

The ISO 2631-1:1997 and underlying directives such as European Directive 2002/44/EC currently provide the only verified limits for health and safety concerning WBV. Accordingly, to fully comprehend the impact of WBV on the incidence of LBP in alpine skiers, additional systematic and/or epidemiological studies are required. Furthermore, various slopes, snow conditions, ski racing disciplines, ages of the skiers/athletes, etc. needed to be investigated in a standardized manner to enable more focused preventive measures. In particular, monitoring the training load of the athletes at highest intensity, as suggested earlier (Spörri et al., 2017), with miniaturized equipment would be beneficial, but the equipment must, of course, comply with the ISO 8041:2005 requirements.

## Conclusions

Here, we show that with all types of alpine skiing examined WBV exceeded health and safety limits, with the more advanced forms such as short swinging or carved turns exceeding these limits by as much as ~30-fold. Thus, alpine skiing, where active participants can train 100–150 days each year with demanding snow conditions and slopes and high loads, appears to be associated with high long-term risks to health. One appropriate preventive measure would be to reduce the number of skiing days and/or at least the number of runs and skiing days involving conditions where WBV are strongest (e.g., with side-skidding). This is particularly important for younger skiers, since many deteriorations of the spine develop early in adolescence (Rachbauer et al., 2001). At the same time, alpine skiing has several positive effects on health (Müller et al., 2011a,b). Therefore, for most recreational skiers, with relatively few skiing days each year, the preventive measures would be to ski on natural (not icy) snow and slopes where employing skiing techniques associated with weaker WBV are possible and safe.

## Author contributions

MS designed the study. MS and JO prepared the equipment for data collection, performed the measurements and prepared the platform for computations. JO performed the statistical analyses. MS, JO, and H-CH performed the data analysis and interpretation. MS wrote the first draft and all authors contributed substantially to and approved the final version.

### Conflict of interest statement

The authors declare that the research was conducted in the absence of any commercial or financial relationships that could be construed as a potential conflict of interest.
